# Effect of Radiological Countermeasures on Subjective Well-Being and Radiation Anxiety after the 2011 Disaster: The Fukushima Health Management Survey

**DOI:** 10.3390/ijerph15010124

**Published:** 2018-01-12

**Authors:** Michio Murakami, Yoshitake Takebayashi, Yoshihito Takeda, Akiko Sato, Yasumasa Igarashi, Kazumi Sano, Tetsuo Yasutaka, Wataru Naito, Sumire Hirota, Aya Goto, Tetsuya Ohira, Seiji Yasumura, Koichi Tanigawa

**Affiliations:** 1 Radiation Medical Science Center for the Fukushima Health Management Survey, Fukushima Medical University, 1 Hikarigaoka, Fukushima 960-1295, Japan; ytake2@fmu.ac.jp (Y.T.); agoto@fmu.ac.jp (A.G.); teoohira@fmu.ac.jp (T.O.); yasumura@fmu.ac.jp (S.Y.); tanigawa@fmu.ac.jp (K.T.); 2 Department of Health Risk Communication, Fukushima Medical University School of Medicine, 1 Hikarigaoka, Fukushima 960-1295, Japan; 3 Graduate School of Environment and Information Sciences, Yokohama National University, 79-7 Tokiwadai, Hodogaya, Yokohama 240-8501, Japan; takeda-yoshihito-tb@ynu.ac.jp; 4 United Nations University Institute for the Advanced Study of Sustainability, 5-53-70 Jingumae, Shibuya, Tokyo 150-8925, Japan; akiko.sato@unu.edu; 5 Graduate School of Humanities and Social Sciences, University of Tsukuba, 1-1-1 Tennoudai, Tsukuba, Ibaraki 305-8571, Japan; vyl03222@nifty.com; 6 Center for Material Cycles and Waste Management Research, National Institute for Environmental Studies, 16-2 Onogawa, Tsukuba, Ibaraki 305-8506, Japan; kamis@mue.biglobe.ne.jp; 7 Research Institute for Geo-Resources and Environment, National Institute of Advanced Industrial Science and Technology, 1-1-1 Higashi, Tsukuba, Ibaraki 305-8567, Japan; t.yasutaka@aist.go.jp; 8 Research Institute of Science for Safety and Sustainability, National Institute of Advanced Industrial Science and Technology, 16-1, Onogawa, Tsukuba, Ibaraki 305-8569, Japan; w-naito@aist.go.jp; 9 Faculty of Informatics, Tokyo City University, 3-3-1, Ushikubonishi, Tsuzuki, Yokohama, Kanagawa 224-8551, Japan; sumire@tcu.ac.jp; 10 Center for Integrated Science and Humanities & International Community Health, Fukushima Medical University, 1 Hikarigaoka, Fukushima, Fukushima 960-1295, Japan; 11 Department of Epidemiology, Fukushima Medical University School of Medicine, 1 Hikarigaoka, Fukushima 960-1295, Japan; 12 Department of Public Health, Fukushima Medical University School of Medicine, 1 Hikarigaoka, Fukushima 960-1295, Japan

**Keywords:** anxiety, Fukushima Daiichi nuclear power plant accident, radiation, risk communication, subjective well-being

## Abstract

After the Fukushima Daiichi Nuclear Power Station accident in 2011, concerns about radiation exposure and decline in subjective well-being have been reported. To tackle these problems, various countermeasures in relation to radiation have been implemented. In this study, we comprehensively evaluated the effects of radiological countermeasures on subjective well-being (e.g., satisfaction with life (SWL) and emotional well-being) and radiation anxiety, through a questionnaire survey targeting Fukushima residents (*N* = 1023). Propensity scores matching was applied to evaluate significant effects of radiological countermeasures on subjective well-being and radiation anxiety. Among the radiological countermeasures, thyroid examination, whole body counter, and air dose monitoring showed the highest proportions of participation, utilization, and useful evaluation, suggesting a high degree of public attention focused on these countermeasures. The basic survey was associated with significant increases in SWL and self-rated health (SH). Thyroid examination was significantly associated with not only a reduction in radiation anxiety but also an increase of emotional stress, suggesting the importance of careful design of system and detailed communication. Food inspection was associated with deterioration in SH. Those who utilized explanatory meetings showed increases in sadness, worry, and radiation anxiety, indicating that additional attention is required of the experts and authorities involved in explanatory meetings.

## 1. Introduction

The Fukushima Daiichi Nuclear Power Station (FDNPS) accident following the Great East Japan Earthquake on 11 March 2011 (hereinafter, the 2011 disaster) generated multiple risks. While radiation exposure has been estimated to be <30 mSv of additional lifetime effective dose [1,2], concerns about radiation risks have not been dispelled among the public [3,4]. Furthermore, an increase in other health risks such as psychological distress and lifestyle diseases has been reported among residents in Fukushima, especially evacuees [5,6,7,8]. Psychological distress was reported to be associated with a higher radiation risk perception [9], and its prevalence has decreased gradually but is still higher than before the 2011 disaster [10]. Murakami et al. [11] developed loss of happy life expectancy (i.e., loss of lifespan that people live with a subjective emotional feeling of well-being due to increase of mortality rates as well as reduction in emotional happiness) as a risk indicator by using objective survival probabilities and a question regarding emotional happiness and reported that the loss of happy life expectancy due to psychological distress in evacuees was approximately one to two orders of magnitude higher than that due to cancer mortality caused by radiation exposure after the 2011 disaster, highlighting the importance of countermeasures to minimize distress following public health emergencies. Similarly, a decline in subjective well-being in residents around the FDNPS was reported after the 2011 disaster [12,13]. In particular, anxiety about radiation was associated with a decline in subjective well-being [14] and a reduction in anxiety could aid in improving the residents’ subjective well-being [15]. The United Nations adopted the 2030 Agenda for Sustainable Development, which targets “good health and well-being” as one of 17 goals for developed and developing countries alike [16], and the improvement of well-being has been recognized as an important issue in environmental health sciences and policy [17]; therefore, the promotion of psychological health and subjective well-being has gained increased importance. The Sendai Framework for Disaster Risk Reduction (2015–2030) also recently highlighted the importance of psychological health and well-being [18].

To tackle the problems posed after the 2011 disaster, various radiological countermeasures have been implemented. Various types of “explanatory meetings” have been held in which authorities or professionals have explained situations to affected people, and time for questions and answers was sometimes provided as dialogue [19,20]. Furthermore, in the context of the aftermath of the 2011 disaster, some radiological countermeasures, especially monitoring of individual doses and radionuclide concentrations in foods, were also used as risk communication tools. Risk information was exchanged to support residents’ decision making on what to eat, where to live, and when to return home [4,20,21,22,23]. These activities identified radiation risks and they also addressed radiation anxiety, comprehensive health risks to individuals, and well-being [20] and were well in accordance with the concept of risk communication, which has been defined by the National Research Council as “an interactive process of exchange of information and opinion among individuals” [24]. For example, whole body counter (WBC), i.e., the monitoring of internal radiation exposure, was first used to measure levels of internal radiation doses for medical interventions but then became a tool for risk communication: the WBC creates the opportunity for experts to discuss radiation directly with individuals and address their concerns [20,25]. In addition to radiation monitoring of foods and air dosages, “individual” dose monitoring has been performed by collecting and providing external radiation exposure data using devices such as glass badges [4,26,27]. The basic survey has been conducted under the Fukushima Health Management Survey (FHMS) and involves the assessment and provision of information regarding 4-monthly external exposure after the 2011 disaster among residents in the Fukushima Prefecture [28,29]. These countermeasures are useful for sharing risk information among the public in addition to promoting protection against radiation. The FHMS has also launched thyroid ultrasound examination for all children who were aged 18 years or younger at the time of the 2011 disaster in Fukushima Prefecture. The FHMS has provided the results to the participants and their families, given the importance of long-term follow-up of all children in the prefecture and the considerable anxiety of their parents [28,30].

To facilitate the implementation of effective countermeasures, it is necessary to comprehensively evaluate their effects; however, these have not been investigated in depth except for a few studies examining differences in radiation anxiety before and after explanatory meetings [19,31]. The assessment of disaster countermeasures will guide the further implementation of health policies in Fukushima, and it might also be useful for global disaster preparedness. Objectives of risk countermeasures depend on each activity and the authorities [32]. Radiological countermeasures after the 2011 disaster have been implemented not only to reduce risk but also to improve overall well-being among affected individuals. A logical step is therefore to investigate the effects of radiological countermeasures on multiple outcomes such as subjective well-being, reduction in radiation anxiety, and radiation risk acceptance, which are often regarded as the objectives of those countermeasures [15,19,31,33,34].

In this study, we first investigated Fukushima residents’ participation in, and utilization and evaluation of the following radiological countermeasures: WBC, food inspection, air dose monitoring, individual dose monitoring, basic survey, thyroid examination, and explanatory meetings. We examined the associations between these countermeasures according to participation (i.e., whether or not respondents participated in the countermeasures) and utilization (i.e., whether or not respondents utilized results from the countermeasures). Secondly, we evaluated the effect of the radiological countermeasures on subjective well-being, a reduction in radiation anxiety, and radiation risk acceptance. We hypothesized that radiological countermeasures would aid in improving the well-being in residents in Fukushima, as the nature of objectives of countermeasures. No previous studies have examined the effects of radiological countermeasures on well-being, so we performed an exploratory analysis of the effects of the various countermeasures. This is the first study to comprehensively assess the effects of various radiological countermeasures on subjective well-being after the 2011 disaster.

## 2. Materials and Methods

### 2.1. Procedures and Participants

Ethical approval for the study was granted by the Fukushima Medical University Ethics Committee (approval number: 2722). We developed the questionnaire for this study and conducted online surveys. Online questionnaires were completed in 22–24 August 2016 by individuals aged from 20 to 69 years in Fukushima Prefecture. Since the objective of this study was to obtain an assessment using the responses provided by the people living in Fukushima Prefecture, we targeted all the people living in the Prefecture. The participant pool consisted of members of the general public who had registered as survey panelists with Macromill (Tokyo, Japan), one of the biggest survey companies in Japan, which had 1.18 million panelists in Japan and eight thousand panelists in Fukushima Prefecture at the time of the survey. The company established target numbers of participants for the online survey and then requested registered members to respond to the questionnaires until this target number of participants was achieved. The reliability and advantages of online surveys have been described previously [34]. Briefly, participants received reward points that could be exchanged for cash and commercial products after the survey. The participants were therefore motivated to complete the questionnaire irrespective of their level of interest in the topic, which can reduce potential bias often occurring in mail surveys or central location testing. Responses were excluded if the response time was short (top 3%), if there was a discrepancy in gender or age between the survey response and register information (±1-year age difference was accepted), or if respondents lived outside Fukushima Prefecture when the questionnaire was completed. In total, 1023 individuals participated in the survey.

Since participant selection was not based on composition ratios for gender or age because of limitations in the number of panelists, we examined the distribution of respondents’ gender and age (Table 1). The gender distribution in the study sample was similar to that among all residents in Fukushima Prefecture in August 2016 [35], while in terms of age, there were fewer respondents in their 60 s.

### 2.2. Questionnaire

The questionnaire collected respondents’ age, gender, and location: Hamadori (east coast of Fukushima Prefecture), Nakadori (central region of Fukushima Prefecture), Aizu (western mountainous region of Fukushima Prefecture), or other prefectures.

#### 2.2.1. Subjective Well-Being, Reduction in Radiation Anxiety, Radiation Risk Perceptions, and Radiation Risk Acceptance

Satisfaction with life (SWL), emotional well-being, current subjective feeling of health (self-rated health [SH]), comparison of current SH with that before the disaster, current anxiety regarding radiation, comparison of current radiation anxiety with that in 2011, radiation risk perception, and acceptance of radiation risk were also assessed.

For SWL, overall satisfaction with life at the present time was measured on a scale from 0 (very unsatisfied) to 10 (very satisfied), in accordance with previous reports [11,12,15]. This questionnaire item was included because it has been used in several studies including the study after the 2011 disaster [11,12,15]. Details about this questionnaire item are described elsewhere [36]. Regarding emotional well-being, respondents were asked to answer “yes” (1) or “no” (0) to the following questions [11,37]: “Did you experience a feeling of enjoyment [happiness, stress, sadness, worry] yesterday?” and “Did you laugh yesterday?” SWL and emotional well-being are indicators of “judgments about feelings” and “emotions”, respectively, within Nettle’s three categorizations of happiness (i.e., emotions, judgments about feelings, and *eudaimonia* (i.e., quality of one’s life in terms of achieving one‘s potential)) [38]. This questionnaire item was included because it was applied in the previous study conducted in Japan after the 2011 disaster [11]. The validity and reliability were also discussed in the previous studies [11,37].

Current SH (i.e., SH at the present time) was measured on a 5-point scale by asking “How is your health condition at the present time?”: 1 (very bad), 2 (bad), 3 (normal), 4 (good), and 5 (very good). Comparison of current SH with that before the disaster (i.e., SH improvement) was measured on a 5-point scale by asking “How did your health condition change compared to that before the disaster?”: 1 (worse compared to before), 2 (somewhat worse compared to before), 3 (no change compared to before), 4 (somewhat better compared to before), and 5 (better compared to before). Current anxiety about radiation was measured on a 4-point scale: 1 (very unworried), 2 (unworried), 3 (worried), and 4 (very worried). Neutral points were not provided in this questionnaire, because we expected participants to choose whether they were (very) unworried or worried. Comparison of current radiation anxiety with that in 2011 (i.e., radiation anxiety reduction) was measured on a 5-point scale: 1 (anxiety increased compared to before), 2 (anxiety somewhat increased compared to before), 3 (no change compared to before), 4 (anxiety somewhat reduced compared to before), and 5 (anxiety reduced compared to before) in accordance with the previous study conducted in Japan after the 2011 disaster [15]. We asked about radiation anxiety reduction because this is an important factor that contributes to SWL [15]. The results from this study also provide an advantage as they enable comparison with the results of the previous study. The comparison of current SH with that before the disaster was new information obtained in this study. A similar questionnaire with the same scale was utilized.

Four types of radiation risk perceptions were assessed: dread, unknown, delayed effects, and genetic effects (Table S1). Dread and unknown risk perceptions are the basis of Slovic’s two psychological dimensions [39] and each were assessed using a single question in the present study to reduce respondents’ burden: “Radiation is intuitively dreaded” [1 (strongly disagree) to 4 (strongly agree)] and “Health risks from radiation are known to science” [1 (strongly agree) to 4 (strongly disagree); reversed scoring was applied]. Delayed and genetic effects were assessed following previous studies [9,40]: “What do you think is the likelihood of damage to your health (e.g., cancer onset) in later life as a result of your current level of radiation exposure?” [1 (very unlikely) to 4 (very likely)] and “What do you think is the likelihood that the health of your future (i.e., as yet unborn) children and grandchildren will be affected as a result of your current level of radiation exposure?” [1 (very unlikely) to 4 (very likely)]. These questionnaire items were often used in Japan after the 2011 disaster and details are discussed elsewhere [41]. Neutral points were not provided in these questionnaires, in accordance with the previous studies [9,34]. Current acceptance of radiation risk after the 2011 disaster was assessed using five categories: “never mind”, “acceptable”, “can’t help but accept”, “unacceptable” and “can’t judge” (Table S2).

#### 2.2.2. Participation, Utilization, and Evaluation of Radiological Countermeasures

Risk information can be utilized even by the people other than those involved in risk communication. For example, people who did not participate in risk communication could hear the results from others who attended or could be informed through local municipality reports or news. By contrast, people who attended the risk communication might not use the findings. Therefore, in this study, respondents were asked to report their participation (i.e., whether or not they participated) in and utilization (i.e., whether or not they utilized results as a reference) of radiological countermeasures. They were also asked to provide a subjective evaluation (i.e., whether or not they highly evaluated [whether or not they perceived each countermeasure was useful]).

The radiological countermeasures assessed in this study included WBC, food inspection, air dose monitoring, individual dose monitoring, basic survey, thyroid examination (specific to the examination under the FHMS), and explanatory meetings. WBC is the monitoring of internal radiation exposure and provides opportunities for the experts and examinees to exchange the individual dose information [20,25]. Food inspection is the measurements of radionuclide concentrations in foods. In the aftermath of the 2011 disaster, a food inspection service was available for residents who wanted to monitor their own foods [21]. Many air dose monitoring posts were launched after the 2011 disaster and the dose levels are displayed and delivered to residents. Basic survey, which has been conducted under FHMS, consists of a questionnaire that asks Fukushima Prefecture residents about their behavior in the first four months after the accident. Responses to the questionnaire have been returned by many residents (see details in ref. [29]). The thyroid examination has been performed under the FHMS to follow the status of thyroid cancer among subjects and to provide the results to the subjects and their family members [28,30]. This examination has targeted all children aged 18 years or younger at the time of the 2011 disaster in Fukushima Prefecture. Explanatory meetings have been implemented in a variey of forms, such as one-way delivery of information and dialogues among stakeholders [19,20]. The explanatory meetings for this study included various types of meetings regarding radiation.

##### Participation

Choices in participation included “the respondent himself/herself participated”, “the family participated”, and “neither the respondent nor the family participated”. In the case of air dose monitoring, participation represented own or family’s measurement or confirmation of doses. For thyroid examination, “did not participate” had two choices: “the respondent or family members are subjects of examination (i.e., all children aged 18 years or younger at the 2011 disaster (who were born between 2 April 1992 and 1 April 2011 and living in Fukushima Prefecture at the 2011 disaster) and those who were born between 2 April 2011 and 1 April 2012 in Fukushima Prefecture)” or “neither the respondent nor the family members are subjects of examination”.

##### Utilization

Choices in utilization included “utilized the results of the respondent and/or their family”, “utilized the results of acquaintances”, or “did not utilize”. For WBC, food inspection, individual dose monitoring, basic survey, and thyroid examination, “utilized the summarized results of local municipalities or prefecture” was also included as a response.

##### Evaluation

Evaluation included six choices: “very useful”, “useful”, “neither”, “not useful”, “not useful at all”, and “don’t know” (Table S3).

#### 2.2.3. Other Questionnaire Items

Respondents also provided the following data: whether or not they trusted the information, presence/absence of advisors or counselors regarding the nuclear accident, life events experienced within a year, employment status, evacuation experience, presence/absence of spouse, children, and grandchildren, educational background (i.e., junior or high-school graduates or university graduates etc.), presence/absence of jobless person within the household, household annual income, educational course (i.e., science course, neither, or humanities course), and attendance of social activities (Table S4). Since trusted information was a key factor for risk perception and radiation anxiety [34,41] and was considered as a covariate, participants were asked about the presence or absence of the following information sources: “national newspapers, TVs, and radios”, “local newspapers, TVs, and radios”, ”magazines”, “central government”, “international organization”, “local municipality”, “explanatory meetings of central government and prefecture”, “direct information from medical professionals”, “direct information from experts”, “books written by experts”, “direct information from family and friends in Fukushima prefecture”, “direct information from family and friends outside Fukushima prefecture”, “on-line information from experts”, and “on-line information from other”. Life events experienced within a year were assessed according to a previous study [42] and included 21 choices (20 events and none above). The 20 events included were: “a change of habitation”, “an increase in new family members”, “independence of family members”, “deterioration of own health”, “hospital admission and discharge”, “deterioration of spouse or family members’ health”, “divorce or separation from spouse”, “bereavement of spouse”, “bereavement of a family member (except spouse), ”bereavement of a familiar friend”, ”deterioration of economic status”, “increase in debt”, “misemployment”, “resignation”, “increase in job burdens”, “loss of social roles”, “increase in troubles with family members”, “an increase of troubles with neighbors or friends”, “trouble with the law”, and “other stressful events”. “An increase in new family members” used in the previous study [42] was excluded to consider only potentially negative life events, such as deterioration of own health and bereavement of spouse.

### 2.3. Data Procedure

There were no missing data. Since six categories of acceptance of radiation risk were not on an ordinal scale, we separated them into two classifications to allow application of the same statistical analysis as used for the other outcomes in agreement with the previous study [34]: “never mind, acceptable, or can’t help but accept” and “unacceptable or can’t judge”.

Participation responses were classified into two categories: “participated” (respondents own/their family members) or “did not participate”. Utilization responses were also classified into two categories: “utilized” (irrespective of own/family members’, acquaintances’, or local municipalities’/prefecture’s results) or “did not utilize”. In other words, when people did not participate in the countermeasure but referred or utilized the results of others, including their family members, and summaries of local municipalities or prefectures, they were regarded as “did not participate” and “utilized”. Since six categories of evaluation were also not ordinal scaled and a proportion of the positive evaluation is a useful indicator for health policy, we classified these into two categories similar to those described above: “very useful or useful” and “neither, not useful, not useful at all, or don’t know”.

Ages were classified into 20 s, 30 s, 40 s, 50 s, and 60 s. Trust in each information type was divided into two responses: absence or presence. Other responses regarding the basic characteristics of the respondents were classified into 2–4 groups as shown in Table S4.

### 2.4. Statistical Analysis

The chi-squared test was used to evaluate differences in the proportion of utilization/evaluation of radiological countermeasures according to participation. For thyroid examination, parents of the children who are the subjects of the examination are considered to have strong anxiety and interests in this examination [28,43]. Analyses were therefore performed by taking into account all respondents (*n* = 1023), as well as only those (*n* = 373) reporting that they themselves or their family members are subjects of examination (i.e., all children aged 18 years or younger at the 2011 disaster). Some subects participated and other did not participate in the examination. The Spearman correlation coefficient ρ was tested among the outcomes to demonstrate whether there were reasonable correlations each other.

To evaluate the effects of radiological countermeasures on multiple outcomes, we matched propensity scores [44] using 1:1 nearest-neighbor matching with a ±0.1 caliper and no replacement. The propensity score matching is a powerful statistical technique for demonstrating the causal effects in observational studies [45]. The propensity score is the conditional probability of assignment to a particular treatment given a vector of observed covariates, and it is useful for reducing selection bias due to all the observed covariates [44]. Samples were first matched to make pairs on the basis of a univariate propensity score estimated from the covariates (e.g., pairs of “participated in WBC” and “did not participate in WBC” with adjustments of covariates). The matched samples were then compared between two classes (i.e., a dependent variable) to evaluate the effects for outcomes. Outcomes included SWL, emotional well-being, SH, SH improvement, radiation anxiety, radiation anxiety reduction, and acceptance of radiation risk. Participation in or utilization of each radiological countermeasure was regarded as the dependent variable. We performed propensity scores matching separately for each dependent variable. Matching involved adjustment for age, gender, location, radiation risk perception, participation in/utilization of other radiological countermeasures (e.g., when participation in WBC was the dependent variable, participation in other radiological countermeasures was used), trust of information, presence/absence of advisors or counselors, experience of life events within a year, employment status, evacuation experience, presence/absence of spouse, children, and grandchildren, educational background, presence/absence of jobless person within the household, household annual income, educational course, and attendance of social activities. Variance inflation factors ranged between 1.14–4.07 (arithmetic mean: 1.72). The number of matched pairs for participation in radiological countermeasures were as follows: WBC, 250 (the average of absolute caliper value: 0.02); food inspection, 192 (0.01); air dose monitoring, 224 (0.02); individual dose monitoring, 230 (0.02); basic survey, 277 (0.02); thyroid examination (all respondents), 178 (0.02); thyroid examination (respondents or their family members being subjects of examination), 56 (0.02); and explanatory meetings, 108 (0.009). Regarding utilization of radiological countermeasures, the number of matched pairs was as follows: WBC, 210 (0.02); food inspection, 175 (0.03); air dose monitoring, 170 (0.02); individual dose monitoring, 184 (0.02); basic survey, 191 (0.02); thyroid examination (all respondents), 162 (0.02); thyroid examination (respondents or their family members being subjects of examination), 55 (0.02); and explanatory meetings, 118 (0.01). The Mann-Whitney U test was used to test differences in outcomes according to participation in/utilization of radiological countermeasures before matching. Wilcoxon’s matched-pairs signed-rank test was used to test differences in outcomes between pairs after matching. 

The propensity score matching was performed using R statistical software [45]. Other analyses were performed using IBM SPSS Statistics 24 (IBM, Armonk, NY, USA).

## 3. Results

### 3.1. Participation in, Utilization of, and Evaluation of Radiological Countermeasures

Table 2 shows the numbers and proportions of respondents who participated in, utilized, and highly evaluated each radiological countermeasure. The participation proportion was highest for thyroid examination (79%; considering respondents or their family members being subjects of examination), followed by WBC (53%), air dose monitoring (52%), and other radiological countermeasures (12–42%). Similarly, the utilization proportion was highest for thyroid examination (72%; respondents or their family members being subjects of examination), and lowest for explanatory meetings (15%). The utilization proportions for other radiological countermeasures ranged from 35% to 52%. The useful evaluation proportion (i.e., proportion of those whose responses were “very useful or useful”) was highest for food inspection (68%) and thyroid examination (64%; respondents or their family members being subjects of examination), followed by air dose monitoring (61%), WBC (54%), and other radiological countermeasures (38–41%). The participation proportion for the basic survey was 31% (respondents themselves) in this study, which is very similar to the actual participation proportion (26%; as of 30 June 2014) [29]. Similarly, the participation proportion for thyroid examination (79%; respondents or their family members being subjects of examination) showed a good agreement with the actual participation proportion (82%; from 9 October 2011 to 31 March 2014) [30]. These results indicate that participant selection biases were small in this study.

Table 3 compares proportions of utilization/useful evaluation for each radiological countermeasure according to participation. Those who participated in each radiological countermeasure showed significantly higher proportions of utilization (71–90%) than did those who did not participate (note that “utilization” for those who did not participate in the radiological countermeasures means utilization of acquaintances’ or local municipalities’/prefecture’s results). Those who did not participate in the thyroid examination showed a relatively higher proportion of utilization (i.e., utilization of results of acquaintances, local municipalities or the prefecture as a reference; 29%) in comparison with other radiological countermeasures (6–26%). Except for thyroid examination (respondents or their family members being subjects of examination), those who participated in each radiological countermeasure also showed significantly higher proportions of evaluation as useful than did those who did not participate. Among the countermeasures, participants showed relatively high useful evaluation for food inspection (79%) and air dose monitoring (72%), followed by thyroid examination (65%).

Before investigating the effects of radiological countermeasures on the multiple outcomes, we confirmed associations of participation between countermeasures and associations of utilization between countermeasures (Table 4). There were significant associations in participation between all countermeasures and in utilization between all countermeasures. Odds ratios for participation ranged from 2.00 (95% confidence interval (CI): 1.47–2.72) for food inspection vs. thyroid examination, to 13.7 (95% CI: 9.16–20.4) for WBC vs. thyroid examination; whereas the odds ratios for utilization ranged from 6.80 (95% CI: 5.16–8.97) for WBC vs. air dose monitoring, to 18.3 (95% CI: 13.1–25.7) for food inspection vs. air dose monitoring. These indicated that those who participated in (or utilized a result of) a countermeasure tended to participate in (or use the results of) other countermeasures.

### 3.2. Effect of Radiological Countermeasures on Subjective Well-Being and Radiation Anxiety

Table 5 shows the arithmetic mean and standard deviation (SD) of scores for each outcome. The distributions are shown in Table S5. SWL scores of 5–8 over 10 were frequently found and the arithmetic mean SWL score was 5.90. This was comparable to the values in Japan (6.46; questionnaire drop-off (i.e., the investigators dropped off the questionnaire sheet at participants’ their home, and then revisited the home to collect the completed questionnaire, national survey) [12] and in Marumori Town, Igu County, Miyagi Prefecture (6.34; mailing method; approximately 50 km northwest of FDNPS) [15]. The arithmetic mean score for subjective happiness in this study was 0.56, which was also comparable to that in Japan (0.59; online questionnaire) [11]. Regarding the reduction in radiation anxiety compared to 2011, the arithmetic mean score was 3.13 on a 5-point scale, and only 36% of respondents reported that their anxiety had decreased (sum of “anxiety reduced compared to before” [8%] and “anxiety somewhat reduced compared to before” [28%]). This value was lower than that of 55% reported in Marumori Town, Igu County, Miyagi Prefecture [15], reflecting that anxiety regarding radiation risk still remained among Fukushima residents.

The Spearman correlation coefficient ρ values are summarized in Table S6. SWL and improvements in SH showed significant correlations with all the other outcomes. Generally, indicators of positive feelings of well-being (i.e., SWL, enjoyment, happiness, laughter, SH, improvements in SH, reduction in radiation anxiety, and acceptance of radiation risk) showed direct correlations with each other and inverse correlations with negative feelings of well-being indicators (i.e., stress, sadness, worry, and radiation anxiety).

**Figure 1:**
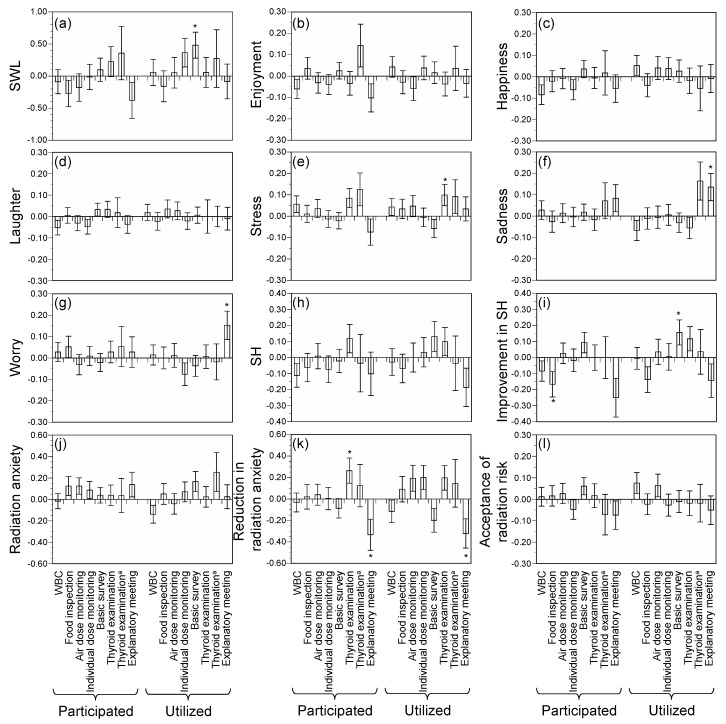
Differences in outcomes according to participation in (or utilization of) radiological countermeasures. Data were adjusted using propensity score matching. The values represent differences in outcomes between pairs of “participated” and “did not participate” or pairs of “utilized” and “did not utilize”. Wilcoxon’s matched-pairs signed-rank test was used to test differences in outcomes between pairs. (**a**) SWL; (**b**) Enjoyment; (**c**) Happiness; (**d**) Laughter; (**e**) Stress; (**f**) Sadness; (**g**) Worry; (**h**) SH; (**i**) Improvement in SH; (**j**) Radiation anxiety; (**k**) Reduction in radiation anxiety; (**l**) Acceptance of radiation risk. Participated: respondents and/or their family participated. WBC: whole body counter; SWL: satisfaction with life; SH: self-rated health. Error bar represents standard error. * *p* < 0.05. ^a^: Respondents or their family members are subjects of examination.

Figure 1 shows differences in outcomes according to participation/utilization of each radiological countermeasure after propensity score matching (i.e., effects of each radiological countermeasure). The results before propensity score matching are shown in Figure S1.

Those who utilized basic surveys had significantly higher SWL than those who did not (Figure 1a, *Z* = 2.331). Those who utilized thyroid examination (all respondents) had a significantly higher stress than those who did not (Figure 1e, *Z* = 1.969). A nonsignificant association in the same direction was observed for those respondents reporting that they or their family members are subjects of examination (*Z* = 1.147), probably owing to weak statistical power. Those who utilized explanatory meetings showed higher sadness and worry levels than those who did not utilize them (Figure 1f,g, *Z* = 2.101 for sadness; *Z* = 2.250 for worry). Those who participated in food inspection showed significantly lower improvements in SH (Figure 1i, *Z* = −2.317) and those who utilized the basic survey showed significantly higher improvements in SH (*Z* = 2.025). Those who participated in thyroid examination (all respondents) showed a significantly larger reduction in radiation anxiety than those who did not participate (Figure 1k, *Z* = 2.262). A nonsignificant association in the same direction was also observed for those respondents reporting that they or their family members are subjects of examination (Figure 1k, *Z* = 0.657). On the contrary, those who participated in or utilized explanatory meetings showed a significantly larger increase in radiation anxiety compared to those who did not (*Z* = −2.275 for participation; *Z* = −2.363 for utilization). For other outcomes (i.e., enjoyment, happiness, laughter, SH, radiation anxiety, and acceptance of radiation risk), there were no significant differences according to participation in/utilization of radiological countermeasures.

We demonstrated the degrees of these effects influenced by countermeasures by normalizing the differences in outcomes that showed significances using the standard deviation of each outcome (Table 6). The differences in outcomes influenced by countermeasures were corresponded to approximately 0.2–0.3 of the standard deviation.

## 4. Discussion

This study reported participation in, and utilization and evaluation of various radiological countermeasures, as well as subjective well-being and radiation anxiety in Fukushima residents five years after the 2011 disaster. Furthermore, we comprehensively assessed the effects of radiological countermeasures on subjective well-being and radiation anxiety with adjustments for associations between these countermeasures.

### 4.1. Implications from Participation in, Utilization of, and Evaluation of Radiological Measures

Among the radiological countermeasures, thyroid examination, WBC, and air dose monitoring showed the highest proportions of participation, utilization, and useful evaluation (Table 2). Those who did not participate in the thyroid examination also showed a relatively high proportion of utilization of thyroid examination results (29%) (Table 3). These results suggest that dose monitoring and thyroid examination played important roles in society in the aftermath of the 2011 disaster. Thyroid examination under the FHMS started because of the importance of long-term follow-up of all children in the prefecture and the considerable anxiety of their parents [28]. This study confirmed the strong public attention focused on thyroid examination. Similarly, high utilization proportions of WBC and air dose monitoring suggest that residents in Fukushima Prefecture used the risk information of doses and these countermeasures are working to exchange or deliver risk information, as previously suggested [20,32]. Food inspection showed lower proportions of participation and utilization than did thyroid examination, WBC, and air dose monitoring, but high values of useful evaluation (68% for all respondents; 79% for those who participated). Food inspection measurements require time, money, and efforts, which probably limit participation and utilization for this countermeasure; however, those who participated in food inspection were satisfied with it. This finding highlights the usefulness of radionuclide measurements, which is consistent with previous reports [21,46].

There were significant associations in participation and utilization between all pairs of radiological countermeasures (Table 4). In general, odds ratios for utilization were higher than those for participation (except in the association between WBC and thyroid examination), indicating that those who utilized the results of a countermeasure strongly tended to use the results of other countermeasures. The opportunities or the system design (e.g., whether countermeasures request people to participate voluntarily or involuntary) differed among the countermeasures, but the utilization of radiological countermeasures reflected respondents’ subjective views more than their participation. Strong associations between radiological countermeasures imply such countermeasures are not independent in terms of participation and utilization; therefore, statistical adjustments between participation in/utilization of radiological countermeasures are necessary to investigate the effects of each countermeasure separately. Although significant differences in multiple outcomes were observed for many radiological countermeasures before propensity score matching (Figure S1), these differences might be attributed to the combined effects of other radiological countermeasures rather than to the effect of the targeted countermeasure only. Propensity score matching was therefore applied to measure the effects of each radiological countermeasure while reducing the effects of other countermeasures and factors (Figure 1).

### 4.2. Implications from Effects of Radiological Measures on Well-Being and Radiation Anxiety

Our study showed that indicators of positive feelings of well-being showed direct correlations with each other and inverse correlations with negative feelings of well-being indicators (Table S6), demonstrating reasonable relationships among outcomes. Overall, among the radiological countermeasures, the basic survey, food inspection, thyroid examination, and explanatory meetings showed significant effects on subjective well-being and radiation anxiety. The differences in outcomes influenced by countermeasures were 0.3 of standard deviation at a maximum, suggesting that the effects were generally smaller for countermeasures than for individual variations. Despite the small effects, the basic survey was associated with an improvement in SWL and SH, which can be interpreted in two ways. Firstly, those who utilized the results of basic surveys knew that the radiation dose was very limited [29] and were satisfied with the dose information. Through this understanding and satisfaction, these individuals possibly had more positive feelings about life and health in general. This is in accordance with the statement by the National Research Council that “risk communication is successful to the extent that it raises the level of understanding of relevant issues or actions and satisfies those involved that they are adequately informed within the limits of available knowledge” [24]. Secondly, since the basic survey requires respondents to fill in relatively complex questionnaires, those with higher levels of subjective well-being might be able to utilize the results of basic survey more easily. This study applied propensity score matching techniques to evaluate the effect of the countermeasures; however, there is still a margin for discussing causal relationships.

Food inspection was associated with deterioration of SH. Radionuclide levels in food were limited in general but some foods (e.g., wild vegetables, mushrooms, and some wild game) exceeded the regulation values especially just after the 2011 disaster [47]. Those who participated in food inspection might think that they could not eat such local foods, especially just after the 2011 disaster, resulting in subjective health deterioration. Another interpretation involves the contrary causal effect, similar to the result of the basic survey: those who perceived health deterioration after the 2011 disaster might tend to participate in food inspection due to health concerns.

Thyroid examination was associated with a reduction in radiation anxiety as well as with an increase in stress at a macro level. The reduction in radiation anxiety is in accordance with an objective of the FHMS [28], but an increase in stress was not expected. The negative effect of thyroid examination on subjective well-being has been regarded as an unintended consequence [48]. Thyroid examination divides individuals into four categories according to their results: A1, no nodule or cyst (51.5% in the first round, performed from 9 October 2011 to 31 March 2014); A2, nodule less than 5.0 mm and/or cyst less than 20.1 mm (47.8% in the first round); B, further examination necessary (nodule ≥ 5.0 mm and/or cyst ≥ 20.1 mm) (0.8% in the first round); and C, urgent need of further examination (0% in the first round). These results are comparable to those of thyroid examinations of children in other prefectures using the same method (42.5% A1, 56.5% A2, 1.0% B, and 0% C) [49]. Approximately half of the individuals were classified as A1 or A2. A2 represents no medical problems; however, individuals and their family tend to have strong concerns about the examination results even in the case of A2 [43]. This suggests that a carefully designed examination system and detailed communication regarding thyroid examination among experts, subjects and their family members are important.

Explanatory meetings were associated with increases in sadness, worry, and radiation anxiety. The increase in radiation anxiety was contrary to previous studies that reported a reduction in radiation anxiety after explanatory meetings [19,31]. Although those who had high radiation anxiety possibly tended to participate in or utilized explanatory meetings, it should be noted that these results were observed after adjustment for radiation risk perception. The gap between this and previous studies is probably due to differences in contents and quality of explanatory meetings. In the questionnaire used in this study, the contents of explanatory meetings were not specified. The effects of explanatory meetings on well-being and radiation anxiety can be influenced by various factors, such as timing, setting, scale, contents, and communicators [20]. Especially just after the 2011 disaster, explanatory meetings with a large number of participants were often held by various “experts”, local municipalities, and the central government. The controversial opinions of “experts” on radiation risks are suggested to have led to confusion among the public [50]. A previous study after the 2011 disaster revealed that meetings with lower numbers of participants were associated with higher anxiety reduction and understanding [19]. The importance of individual-level communication and community involvement was highlighted and applied after the Chernobyl accident [51,52]. Masunaga et al. [53] reported radiation anxiety affected poor mental health status among younger generation around Chernobyl and also highlighted the importance of health risk communication. Empirically, risk-communication activities in Fukushima also shifted gradually to one-to-one or small-group communications [20]. The finding regarding explanatory meetings obtained in this study is consistent with recent trends in risk communication in Fukushima and the lessons learned from the 2011 disaster, and requires the attention of experts and authorities involved in explanatory meetings.

### 4.3. Limitations and Future Perspectives

This study had some limitations. Firstly, there were potential participant biases, which arose from the questionnaire method (*n* = 1023 and online survey). These were sources of uncertainty in this study; however, we conducted an online survey in which respondents received reward points as a motivation to participate irrespective of their interest in the topic. We expected to collect representative participants from Fukushima Prefecture to a great extent, so this method was potentially effective for reducing biases. In fact, participation proportions in the basic survey and thyroid examination were consistent with actual values. Secondly, we used one item measures in the questionnaires to lessen the burden on the participants; this potentially reduced reliability. We confirmed reasonable and significant correlations among the outcomes (Table S5); however, further replicated questionnaires that incorporate multiple item measures are necessary to reduce uncertainty. Thirdly, respondents assessed changes in radiation anxiety and SH retrospectively; therefore, it is possible that recall bias occurred. The recall bias is likely to occur among people who strongly perceived a reduction in radiation anxiety and deterioration of SH; therefore, the results found in this study might be overestimated. Fourthly, we did not evaluate cost-effectiveness of the radiological countermeasures. Fifthly, we targeted the residents in Fukushima Prefecture; however, the state of well-being was considered worse among evacuees. Because of the limited number of samples, we could not evaluate the effects of countermeasures among the people who had been forced to evacuate. Further studies are necessary to focus on these affected people. Sixthly, the effects of radiological countermeasures possibly change dynamically, and continuous surveys for monitoring are therefore needed. Seventhly, as this was a cross-sectional study, causal relationships could not be inferred despite using propensity score matching. It is necessary to carry out a cohort study to more firmly examine causal relationships and influencing factors. The cohort study has another advantage as it allows monitoring of the changes in effects over time and will guide us in judging how to implement countermeasures.

## 5. Conclusions

This is the first study to assess the effects of various radiological countermeasures on subjective well-being and radiation anxiety after the 2011 disaster. The principal conclusions derived from the present study are as follows:

- Among radiological countermeasures, thyroid examination, WBC, and air dose monitoring showed higher proportions of participation, utilization, and evaluation, suggesting a high level of public attention.
- Overall, the effects were generally smaller for countermeasures than for individual variations. The basic survey was associated with an improvement of SWL and SH. Thyroid examination was associated with not only a reduction in radiation anxiety but also an increase in stress. Those who participated in food inspection showed a lower improvement in SH. Those who utilized explanatory meetings showed increases in sadness, worry, and radiation anxiety. For a further assessment of causal relationships, continuous surveys with a cohort design are necessary.

## Figures and Tables

**Table 1:** Distributions of participants in this study and in the general population of Fukushima Prefecture in August 2016.

	Men	Women	20 s	30 s	40 s	50 s	60 s
This study	48%	52%	13%	25%	28%	20%	13%
General population	52%	48%	14%	18%	21%	21%	26%

**Table 2:** Numbers and proportions of those who participated in, utilized, and highly evaluated radiological countermeasures.

	N	Participated ^a^	Participated ^b^	Utilized	Evaluated
WBC	1023	31%	53%	46%	54%
Food inspection	1023	9%	22%	41%	68%
Air dose monitoring	1023	34%	52%	52%	61%
Individual dose monitoring	1023	15%	35%	36%	41%
Basic survey	1023	31%	42%	35%	38%
Thyroid examination	1023	1%	29%	33%	53%
Thyroid examination ^c^	373	4%	79%	72%	64%
Explanatory meeting	1023	7%	12%	15%	39%

WBC: whole body counter; ^a^: Respondents themselves participated; ^b^: Respondents and/or their family participated; ^c^: Respondents or their family members are subjects of examination.

**Table 3:** Differences in proportions of utilization or evaluation for each radiological countermeasure between the participation and non-participation.

		Utilized (%)			Evaluated (%)		
	Participation ^a^	Arithmetic Mean	SE		Arithmetic Mean	SE	
WBC	Did not participate	15%	2%	***	47%	2%	***
	Participated	74%	2%		61%	2%	
Food inspection	Did not participate	26%	2%	***	65%	2%	***
	Participated	90%	2%		79%	3%	
Air dose monitoring	Did not participate	11%	1%	***	49%	2%	***
	Participated	88%	1%		72%	2%	
Individual dose monitoring	Did not participate	11%	1%	***	35%	2%	***
	Participated	81%	2%		51%	3%	
Basic survey	Did not participate	10%	1%	***	34%	2%	**
	Participated	71%	2%		44%	2%	
Thyroid examination	Did not participate	13%	1%	***	48%	2%	***
	Participated	84%	2%		65%	3%	
Thyroid examination ^b^	Did not participate	29%	5%	***	59%	6%	ns
	Participated	84%	2%		65%	3%	
Explanatory meeting	Did not participate	6%	1%	***	38%	2%	*
	Participated	85%	3%		47%	4%	

SE: standard error. WBC: whole body counter. ns: not significant; * *p* < 0.05; ** *p* < 0.01; *** *p* < 0.001. ^a^: Respondents and/or their family participated; ^b^: Respondents or their family members are subjects of examination.

**Table 4:** Associations of participation between countermeasures and associations of utilization between countermeasures. Values represent odds ratios (95% confidence intervals).

	Food Inspection	Air Dose Monitoring	Individual Dose Monitoring	Basic Survey	Thyroid Examination	Explanatory Meeting
Participated ^a^						
WBC	3.30 (2.38–4.57)	4.20 (3.23–5.45)	5.73 (4.26–7.71)	4.40 (3.36–5.77)	13.7 (9.16–20.4)	4.73 (2.95–7.57)
Food inspection	-	10.1 (6.62–15.3)	3.95 (2.91–5.37)	3.33 (2.45–4.52)	2.00 (1.47–2.72)	3.27 (2.22–4.82)
Air dose monitoring	-	-	5.80 (4.32–7.79)	5.17 (3.93–6.80)	3.14 (2.35–4.21)	4.92 (3.08–7.88)
Individual dose monitoring	-	-	-	3.40 (2.60–4.44)	4.61 (3.46–6.15)	4.96 (3.33–7.41)
Basic survey	-	-	-	-	4.81 (3.59–6.44)	4.40 (2.92–6.65)
Thyroid examination	-	-	-	-	-	5.65 (3.81–8.37)
Utilized						
WBC	8.70 (6.53–11.6)	6.80 (5.16–8.97)	7.70 (5.74–10.3)	8.15 (6.05–11.0)	9.44 (6.91–12.9)	9.11 (5.73–14.5)
Food inspection	-	18.3 (13.1–25.7)	9.39 (6.99–12.6)	8.24 (6.16–11.0)	7.05 (5.27–9.43)	9.01 (5.88–13.8)
Air dose monitoring	-	-	14.5 (10.3–20.6)	10.8 (7.78–15.0)	7.00 (5.13–9.55)	8.99 (5.47–14.8)
Individual dose monitoring	-	-	-	10.5 (7.75–14.1)	8.98 (6.67–12.1)	8.67 (5.81–12.9)
Basic survey	-	-	-	-	10.5 (7.75–14.2)	8.18 (5.51–12.1)
Thyroid examination	-	-	-	-	-	12.1 (7.94–18.3)

WBC: whole body counter. ^a^: Respondents and/or their family participated.

**Table 5:** Arithmetic mean and standard deviation (SD) of scores for each outcome. Values in square brackets represent ranges of scales.

	Arithmetic Mean	SD
SWL [0–10]	5.90	2.21
Enjoyment [0–1]	0.54	0.50
Happiness [0–1]	0.56	0.50
Laughter [0–1]	0.81	0.39
Stress [0–1]	0.74	0.44
Sadness [0–1]	0.30	0.46
Worry [0–1]	0.59	0.49
SH [1–5]	3.13	0.86
Improvement in SH [1–5]	2.73	0.74
Radiation anxiety [1–4]	2.45	0.87
Reduction in radiation anxiety [1–5]	3.13	1.02
Acceptance of radiation risk [0–1]	0.52	0.50

SWL: satisfaction with life; SH: self-rated health.

**Table 6:** Effects of countermeasures on outcomes. The differences in outcomes according to radiological countermeasures were regarded as effects. The values were normalized using the standard deviation (SD) of the scores for each outcome.

Countermeasures	Outcomes	Effects (Normalized by SD)
Basic survey (utilization)	SWL	0.22
Basic survey (utilization)	Improvement in SH	0.21
Food inspection (participation)	Improvement in SH	−0.22
Thyroid examination (utilization)	Stress	0.22
Thyroid examination (participation)	Reduction in radiation anxiety	0.26
Explanatory meeting (utilization)	Sadness	0.29
Explanatory meeting (utilization)	Worry	0.31
Explanatory meeting (participation)	Reduction in radiation anxiety	−0.33
Explanatory meeting (utilization)	Reduction in radiation anxiety	−0.32

SWL: satisfaction with life; SH: self-rated health.

## Supplementary Materials

The following are available online at www.mdpi.com/1660-4601/15/1/124/s1, Figure S1: Differences in outcomes according to participation in/utilization of radiological countermeasures before propensity score matching; Table S1: Arithmetic mean, standard deviation (SD) and distributions of radiation risk perceptions; Table S2: Distributions of risk acceptance; Table S3: Distributions of evaluation for each radiological countermeasure; Table S4: Basic characteristics of respondents; Table S5: Distributions of each outcome; Table S6: Spearman’s correlation matrix among outcomes.
