# Supplementary material for: Effect of Radiological Countermeasures on Subjective Well-Being and Radiation Anxiety after the 2011 Disaster: The Fukushima Health Management Survey
Source: Int J Environ Res Public Health. 2018 Jan 12;15(1):124. doi: 10.3390/ijerph15010124 (PMC5800223; doi:10.3390/ijerph15010124)
Supplement: Supplementary file 1 [file ijerph-15-00124-s001.pdf]

Table S1. Arithmetic mean, standard deviation (SD) and distributions of radiation risk perceptions.

	Arithmetic mean	SD	Distributions			
			1	2	3	4
Dread [1-4]	3.03	0.76	3%	18%	52%	27%
Unknown [1-4]	2.66	0.78	7%	32%	49%	12%
Delayed effects [1-4]	2.67	0.81	7%	33%	45%	15%
Genetic effects [1-4]	2.69	0.85	8%	33%	42%	18%

Table S2. Distributions of risk acceptance.

	Distributions
Never mind	19%
Acceptable	16%
Can't help but accept	17%
Unacceptable	16%
Can't judge	31%

Table S3. Distributions of evaluation for each radiological countermeasure.

	Very useful	Useful	Neither	Not useful	Not useful at all	Don’t know
WBC	10%	44%	25%	10%	5%	6%
Food inspection	21%	47%	18%	7%	4%	3%
Air dose monitoring	16%	46%	20%	9%	6%	4%
Individual dose monitoring	7%	34%	33%	12%	7%	6%
Basic survey	7%	31%	34%	13%	10%	6%
Thyroid examination	12%	41%	29%	8%	6%	5%
Thyroid examination[a]	17%	47%	23%	8%	4%	2%
Explanatory meeting	6%	33%	36%	10%	8%	7%

WBC: whole body counter.

a: Respondents or their family members are subjects of examination.

Table S4. Basic characteristics of respondents.

		N (%)
Age	20s	137 (13%)
	30s	260 (25%)
	40s	291 (28%)
	50s	203 (20%)
	60s	132 (13%)
Gender	Men	488 (48%)
	Women	535 (52%)
Employment status	Company employees etc.[a]	499 (49%)
	Self-employed	78 (8%)
	Other[b]	446 (44%)
Location	Hamadori	228 (22%)
	Nakadori	663 (65%)
	Aizu	132 (13%)
Evacuation	Presence of evacuation experience	210 (21%)
	Absence of evacuation experience	813 (79%)
Spouse	Presence of spouse	664 (65%)
	Absence of spouse	359 (35%)
Children	Presence of children	619 (61%)
	Absence of children	404 (39%)
Grandchildren	Presence of grandchildren	92 (9%)
	Absence of grandchildren	931 (91%)
Educational background	Junior or high-school graduates	473 (46%)
	University graduates etc.	550 (54%)
Educational course	Science course[c]	308 (30%)
	Neither	240 (23%)
	Humanities course[d]	475 (46%)
Jobless person within the household	Presence of a jobless person	122 (12%)
	Absence of a jobless person	841 (82%)
	Do not want to answer	60 (6%)
Household annual income	<3 million JPY	275 (27%)
	3 to <6 million JPY	314 (31%)
	≥6 million JPY	242 (24%)
	Do not want to answer	192 (19%)
Social activities	Paritipated	188 (18%)
	Not participated	835 (82%)
Trust of information	National newspapers, TVs, and radios	302 (30%)
	Local newspapers, TVs, and radios	380 (37%)
	Magazines	49 (5%)
	Central government	163 (16%)
	International organization	172 (17%)
	Local municipality	218 (21%)
	Explanatory meetings of central government and prefecture	90 (9%)
	Direct information from medical professionals	197 (19%)
	Direct information from experts	180 (18%)
	Books written by experts	94 (9%)
	Direct information from family and friends in Fukushima prefecture	64 (6%)
	Direct information from family and friends outside Fukushima prefecture	24 (2%)
	On-line information from experts	149 (15%)
	On-line information from other	58 (6%)
	Do not trust either above	309 (30%)
Advisors or counselors	Presence of advisors or counselors	676 (66%)
	Absence of advisors or counselors	347 (34%)
Life events within a year	Presence of life events	635 (62%)
	Absence of life events	388 (38%)

a: full-time non-management; management; company executives and managers; dispatch and contract employees; civil servants and non-profit organization employees; teachers and lecturers; health professionals; and other professionals.
b: part-time, casual, and sideline workers; home duties; students; jobless, retired,etc.
c: “Science course” and “science course chosen from between science course and humanities course”.
d: “Humanities course” and “humanities course chosen from between science course and humanities course”.

Table S5. Distributions of each outcome.

	Score										
	0	1	2	3	4	5	6	7	8	9	10
SWL	3%	1%	4%	8%	5%	18%	14%	21%	18%	4%	3%
Enjoyment	46%	54%	-	-	-	-	-	-	-	-	-
Happiness	44%	56%	-	-	-	-	-	-	-	-	-
Laughter	19%	81%	-	-	-	-	-	-	-	-	-
Stress	26%	74%	-	-	-	-	-	-	-	-	-
Sadness	70%	30%	-	-	-	-	-	-	-	-	-
Worry	41%	59%	-	-	-	-	-	-	-	-	-
SH	-	2%	18%	50%	24%	6%	-	-	-	-	-
Improvement of SH	-	6%	26%	60%	7%	2%	-	-	-	-	-
Radiation anxiety	-	14%	40%	35%	12%	-	-	-	-	-	-
Reduction of radiation anxiety	-	7%	17%	40%	28%	8%	-	-	-	-	-
Acceptance of radiation risk	48%	52%	-	-	-	-	-	-	-	-	-

SWL: satisfaction with life; SH: self-rated health.

Table S6. Spearman's correlation matrix among outcomes.

	Enjoyment	Happiness	Laughter	Stress	Sadness	Worry	SH	Improvements in SH	Radiation anxiety	Reduction in radiation anxiety	Acceptance of radiation risk
SWL	0.37 ***	0.43 ***	0.26 ***	-0.14 ***	-0.14 ***	-0.23 ***	0.44 ***	0.32 ***	-0.13 ***	0.15 ***	0.15 ***
Enjoyment	-	0.74 ***	0.43 ***	0.00 ns	0.00 ns	-0.05 ns	0.29 ***	0.23 ***	0.01 ns	0.04 ns	0.06 ns
Happiness	-	-	0.37 ***	0.01 ns	-0.04 ns	-0.03 ns	0.31 ***	0.22 ***	0.02 ns	0.02 ns	0.04 ns
Laughter	-	-	-	0.04 ns	-0.10 **	-0.03 ns	0.23 ***	0.17 ***	-0.03 ns	0.04 ns	0.07 *
Stress	-	-	-	-	0.28 ***	0.46 ***	-0.13 ***	-0.15 ***	0.10 **	-0.01 ns	0.01 ns
Sadness	-	-	-	-	-	0.41 ***	-0.16 ***	-0.11 ***	0.11 ***	-0.03 ns	-0.01 ns
Worry	-	-	-	-	-	-	-0.19 ***	-0.20 ***	0.12 ***	-0.03 ns	-0.01 ns
SH	-	-	-	-	-	-	-	0.55 ***	-0.22 ***	0.23 ***	0.16 ***
Improvements in SH	-	-	-	-	-	-	-	-	-0.25 ***	0.21 ***	0.11 ***
Radiation anxiety	-	-	-	-	-	-	-	-	-	-0.39 ***	-0.34 ***
Reduction in radiation anxiety	-	-	-	-	-	-	-	-	-	-	0.21 ***

SWL: satisfaction with life; SH: self-rated health. ns: not significant; *: $P < 0.05$; **: $P < 0.01$; ***: $P < 0.001$.

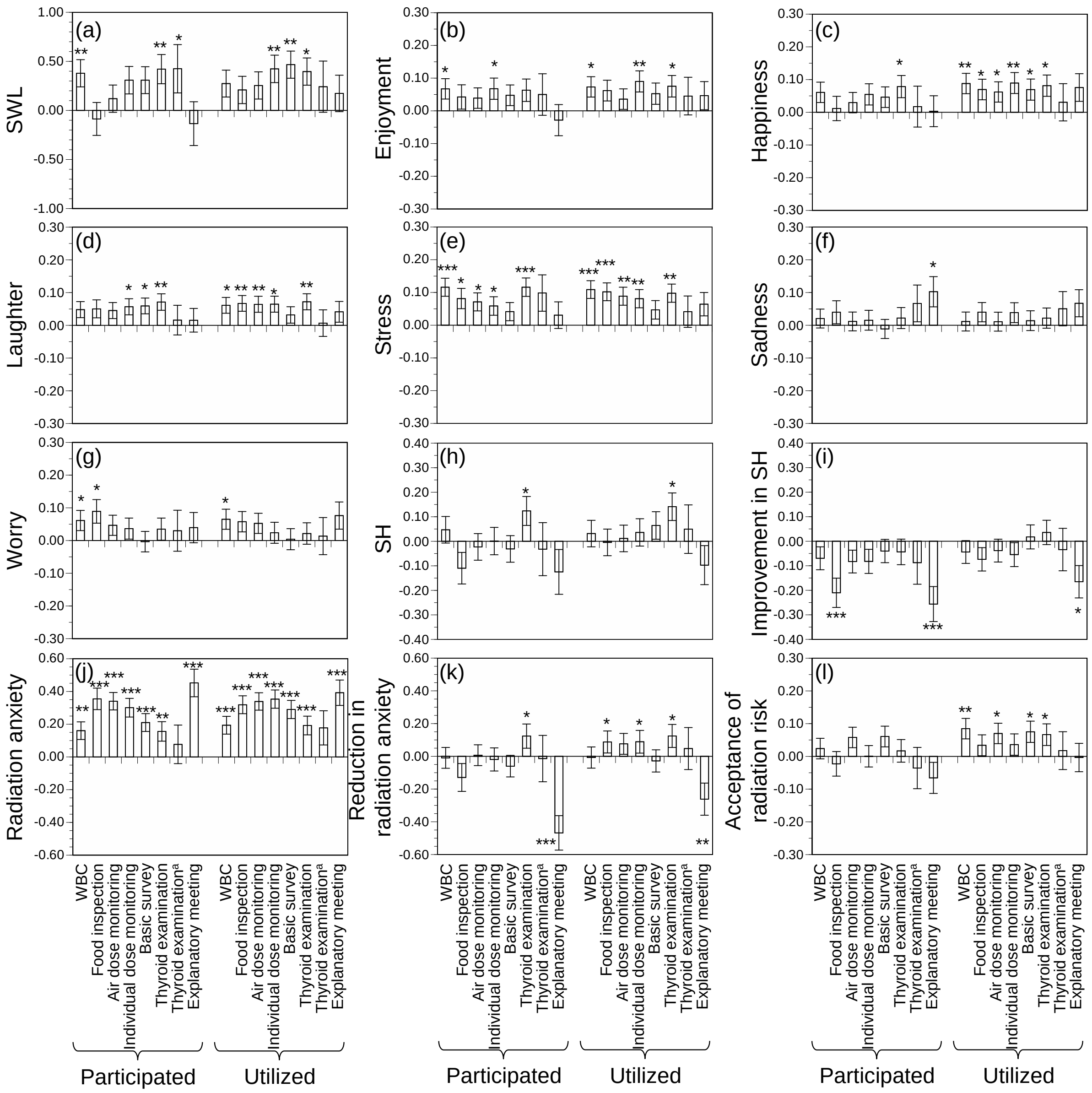

Figure S1. Differences in outcomes according to participation in/utilization of radiological measures before propensity score matching. The values represent differences in outcomes between “participated” and “did not participate” or between “utilized” and “did not utilize.” The Mann-Whitney U test was used to test differences in outcomes according to participation in/utilization of radiological countermeasures. (a) SWL; (b) Enjoyment; (c) Happiness; (d) Laughter; (e) Stress; (f) Sadness; (g) Worry; (h) SH; (i) Improvement in SH; (j) Radiation anxiety; (k) Reduction in radiation anxiety; (l) Acceptance of radiation risk. Participated: respondents and/or their family participated. WBC: whole body counter; SWL: satisfaction with life; SH: self-rated health. Error bar represents standard error.*: $P < 0.05$; **: $P < 0.01$; ***: $P < 0.001$. [a]: Respondents or their family members are subjects of examination..
